# Supplementary material for: Bartonella taylorii: A Model Organism for Studying Bartonella Infection in vitro and in vivo
Source: Front Microbiol. 2022 Jul 15;13:913434. doi: 10.3389/fmicb.2022.913434 (PMC9336547; doi:10.3389/fmicb.2022.913434)
Supplement: Supplementary file 1 [file Data_Sheet_1.pdf]

Supplementary Material

1.1 Supplementary Figures

Figure S1 (related to Figure 1)

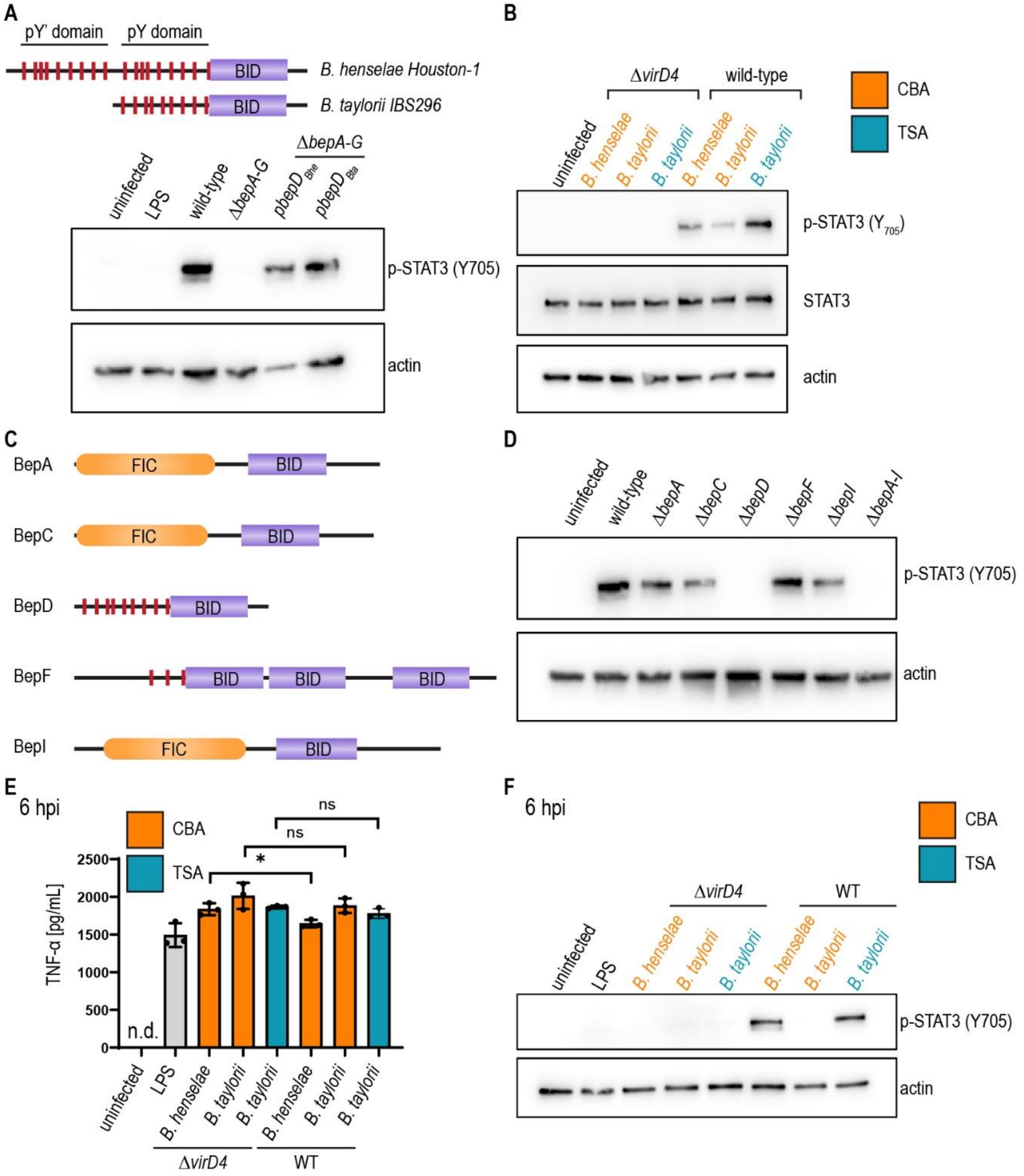

Supplementary Figure S1: BepD$_{Bta}$ ortholog of *B. taylorii* activates STAT3. (A) BepD domain architecture of *B. henselae* Houston-1 and *B. taylorii* IBS296. *B. henselae* harbors 18 tyrosine

residues (red) embedded within the two pY' and pY domains. BepD of *B. taylorii* contains only the pY domain with 9 tyrosine residues. JAWS II dendritic cells were infected at MOI 50 with *B. henselae* wild-type, the Bep-deficient strain *ΔbepA-G*, its $BepD_{Bhe}$-expressing derivative *ΔbepA-G* $pbepD_{Bhe}$ or its $BepD_{Bta}$-expressing derivative *ΔbepA-G* $pbepD_{Bta}$. At 6 hpi, cells were harvested, lysed, and analyzed by immunoblot with specific antibodies against phosphorylated STAT3 (Y705) and actin. (B) Data contributes to figure 1A-D. JAWS II cells were infected at MOI 50 for 24 h with the wild-type or the *ΔvirD4* mutant of *B. henselae* or *B. taylorii* grown on CBA (orange) or TSA (blue). Cells were harvested, lysed and analyzed by immunoblot using specific antibodies against p-STAT3 (Y705), STAT3 and actin. (C) Domain architecture of the Bep repertoire present in *B. taylorii*. FIC domains are displayed in orange, BID domains shown in purple and phosphorylation motifs are shown as red, vertical lines. (D) JAWS II dendritic cells were infected at MOI 50 with *B. taylorii* wild-type, the Bep-deficient strain *ΔbepA-I* or single-*bep* deletions. At 6 hpi cells were harvested, lysed and analyzed by immunoblot for phosphorylated STAT3 (Y705) and actin. (E) JAWS II cells were infected at MOI 50. During the last two hours of infection, cells were treated with 100 ng/mL LPS. 6 hpi supernatant was harvested and TNF-α concentration was assessed by ELISA. (F) Cells in (E) were analyzed by Western Blot for phosphorylated STAT3 (Y705). Actin was used as loading control. Data was acquired by pooling three technical replicates and performed in three independent biological experiments. Data was analyzed using one-way ANOVA with multiple comparisons (Tukey's multiple comparison test), ns = not significant, * $p < 0.05$, FIC = filamentation induced by cyclic AMP; BID = *Bartonella* effector protein intracellular delivery

Figure S2 (related to Figure 2)

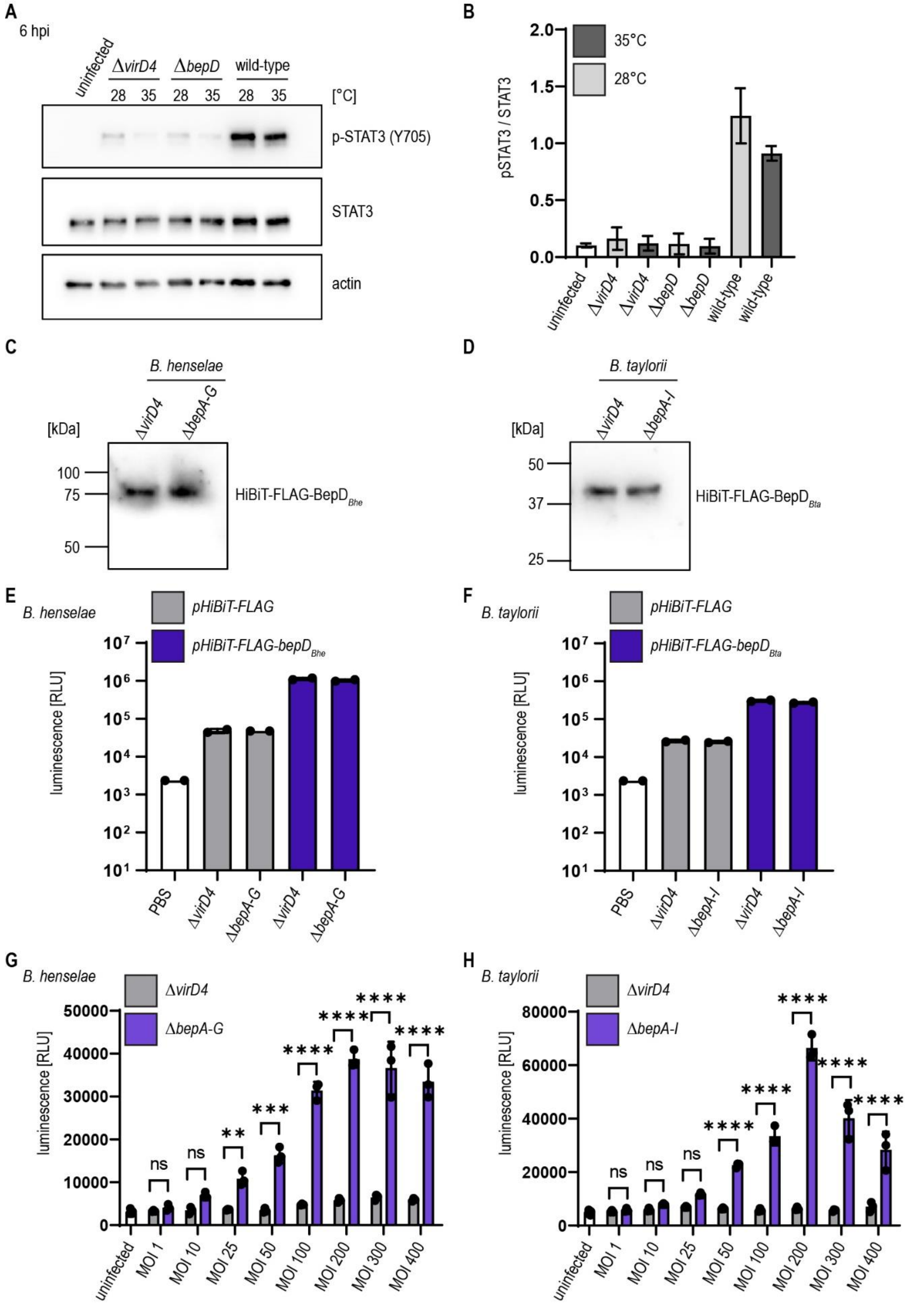

Supplementary Figure S2: BepD$_{\textit{Bhe}}$ and BepD$_{\textit{Bta}}$ are translocated inside RAW macrophages in a *virD4*- and dose-dependent manner. (A) RAW 264.7 macrophages were infected at MOI 50 with *B. taylorii* wild-type, the BepD-deficient mutant *ΔbepD* or the translocation-deficient mutant *ΔvirD4*. Cell lysates were analyzed by Western Blot for phosphorylated STAT3 (Y705), STAT3 and actin. Immunoblot analyzing cell lysates of cells infected for 6 h with bacteria grown at 28°C (light grey) or 35°C (dark grey). (B) Quantification of pSTAT3 signal over STAT3 control of 3 independent immunoblots as shown in (A). (C-D) Immunoblot using specific antibody against the FLAG-epitope. The calculated molecular mass of (C) HiBiT-FLAG-BepD$_{\textit{Bhe}}$ is 63.6 kDa and the calculated molecular mass of (D) HiBiT-FLAG-BepD$_{\textit{Bta}}$ is 42.6 kDa. (E-F) The interaction of HiBiT-FLAG (grey), HiBiT-FLAG-BepD$_{\textit{Bhe}}$ or HiBiT-FLAG-BepD$_{\textit{Bta}}$ (both shown in blue) with LgBiT was tested using the Nano-Glo HiBiT lytic detection system. Lysed bacteria were supplemented with the purified LgBiT protein and the substrate and luminescence measured using the Synergy H4 plate reader for (E) *B. henselae* or (F) *B. taylorii*. (G) RAW LgBiT macrophages were infected with *B. henselae ΔbepA-G* (blue) or *ΔvirD4* (grey) containing *pHiBiT-FLAG-bepD*$_{\textit{Bhe}}$ for 24 h with the indicated MOIs, washed and supplemented with the NLuc substrate. Luminescence was measured in the Synergy H4 plate reader. (H) RAW LgBiT macrophages were infected with *B. taylorii ΔbepA-I* (blue) or *ΔvirD4* (grey) containing *pHiBiT-FLAG-bepD*$_{\textit{Bta}}$ and luminescence measured after 24 hpi. Data was analyzed using one-way ANOVA with multiple comparisons (Tukey's multiple comparison test), ns = not significant, ** $p < 0.01$, *** $p < 0.001$, **** $p < 0.0001$

1.2 Supplementary Tables

Supplementary Table S1. List and construction of all bacterial strains of this study

Strain	Genotype	Reference/Source	Identifier/Description
Escherichia coli			
Novablue	*endA1 hsdR17* (r_{K12}^{-} m_{K12}^{+}) *supE44 thi-1 recA1 gyrA96 relA1 lac F'[proA^{+}B^{+} lacI^{q}ZΔM15::Tn10]*	Novagen	Standard cloning strain
HST08	*F-, endA1, supE44, thi-1, recA1, relA1, gyrA96, phoA, Φ80d lacZΔ M15, Δ (lacZYA - argF) U169, Δ (mrr - hsdRMS - mcrBC), ΔmcrA, λ-*	Takara	Standard cloning strain
JKE201	MFDpir Δ*mcrA* Δ*(mrr-hsdRMS-mcrBC) aac(3)IV::lacI^{q}*	(Harms et al., 2017b)	derivative of MFDpir lacking EcoKI, the three type IV restriction systems, restored gentamicin sensitivity, harboring *lacI^{q}* allele
Bartonella henselae			
B. henselae Houston-1	*rpsL*	(Schmid et al., 2004)	RSE247, spontaneous SmR strain of *B. henselae* ATCC49882T, serving as wild-type
	rpsL pCD366	(Quebatte et al., 2010)	MQB1610; RSE247 containing pCD366
	rpsL pAH196	(Harms et al., 2017b)	MQB1612; RSE247 containing pAH196_Bhe
	rpsL Δ*virD4*	(Schulein et al., 2005)	GS0221; virD4 deletion mutant, derivative of RSE247
	rpsL Δ*virD4 / pHiBiT-FLAG*	This study	KFB286; GS0221 containing pKF059
	rpsL Δ*virD4 / pHiBiT-FLAG-bepD$_{Bhe}$*	This study	MOB120; GS0221 containing pMO006
	rpsL Δ*bepA-G*	(Schulein et al., 2005)	MSE150; bepA-bepG deletion mutant, derivative of RSE247

	rpsL ΔbepA-G / pbepD$_{Bhe}$	(Schulein et al., 2005)	PG4D03; MSE150 containing pPG104
	rpsL ΔbepA-G / pbepD$_{Bta}$	(Sorg et al., 2020)	LUB242; MSE150 containing pLU058
	rpsL ΔbepA-G / pHiBiT-FLAG	This study	KFB276; MSE150 containing pKF059
	rpsL ΔbepA-G / pHiBiT-FLAG-bepD$_{Bhe}$	This study	MOB121; MSE150 containing pMO006
Bartonella taylorii			
B. taylorii IBS296 Sm^R	*rpsL*	(Sorg et al., 2020)	KFB030, spontaneous SmR strain of *B. taylorii IBS296*, serving as wild-type, derivative of LUB046
	rpsL pCD366	This study	KFB266; KFB030 containing pCD366
	rpsL pAH196_Btay	(Harms et al., 2017b)	KFB063; LUB046 containing pAH196_Btay
	rpsL ΔvirD4	This study	KFB146; *virD4* deletion mutant of KFB030
	rpsL ΔvirD4 / pHiBiT-FLAG	This study	KFB291, KFB146 containing pKF059
	rpsL ΔvirD4 / pHiBiT-FLAG-bepD$_{Bta}$	This study	KFB233, KFB146 containing pKF027
	rpsL ΔbepA	This study	KFB068; *bepA* deletion mutant of KFB030
	rpsL ΔbepC	This study	KFB085, *bepC* deletion mutant of KFB030
	rpsL ΔbepD	This study	KFB070; *bepD* deletion mutant of KFB030
	rpsL ΔbepF	This study	KFB097; *bepF* deletion mutant of KFB030
	rpsL ΔbepI	This study	KFB101; *bepI* deletion mutant of KFB030
	rpsL ΔbepA-I	This study	KFB072; *bepA-bepI* deletion mutant of KFB030
	rpsL ΔbepA-I / pHiBiT-FLAG	This study	KFB287; KFB072 containing pKF059

	rpsL ΔbepA-I / pHiBiT-FLAG-bepD$_{Bta}$	This study	KFB263; KFB072 containing pKF027

Supplementary Table S2: List of plasmids used in this study

**Plasmid**	**Backbone**	**Description**	**Reference/Source**
pCD366		RSF1010 derivative encoding promoterless gfpmut2	(Dehio et al., 1998)
pAH196_Bhe	pCD366	pCD366 with PvirB2 of *B. henselae* ahead of gfpmut2	(Harms et al., 2017b)
pAH196_Btay	pCD366	pCD366 with PvirB2 of *B. taylorii* ahead of gfpmut2	(Harms et al., 2017b)
pTR1000		*Bartonella* suicide plasmid with *kanR* / *rpsL* double-selectable cassette for scarless deletions	(Schulein and Dehio, 2002)
pKF001	pTR1000	pTR1000 with homology sites to delete *bepA* of *Bartonella taylorii* (homology regions amplified separately and then fused by SOEing PCR)	This study
pKF002	pTR1000	pTR1000 with homology sites to delete *bepD* of *Bartonella taylorii* (homology regions amplified separately and then fused by SOEing PCR	This study
pKF003	pTR1000	pTR1000 with homology sites to delete *bepC-I* of *Bartonella taylorii* (homology regions amplified separately and then fused by SOEing PCR)	This study
pKF005	pTR1000	pTR1000 with homology sites to delete *bepC* of *Bartonella taylorii* (homology regions amplified separately and then fused by SOEing PCR	This study
pKF006	pTR1000	pTR1000 with homology sites to delete *bepF* of *Bartonella taylorii* (homology regions amplified separately and then fused by SOEing PCR	This study
pKF007	pTR1000	pTR1000 with homology sites to delete *bepI* of *Bartonella taylorii* (homology regions amplified separately and then fused by SOEing PCR	This study
pKF008	pTR1000	pTR1000 with homology sites to delete *virD4* of *Bartonella taylorii* (homology regions amplified separately and then fused by SOEing PCR	This study
pBZ485		new *E. coli* / *Bartonella* shuttle vector based on pCD341 with P*lac*(MQ5); RP4 *oriT*	(Harms et al., 2017a)
pKF059	pBZ485	Derivative of pBZ485, encodes for HiBiT::FLAG	This study
pKF027	pBZ485	Derivative of pBZ485, encodes for HiBiT::FLAG *Bta* BepD fusion protein	This study
pMO006	pBZ485	Derivative of pBZ485, encodes for HiBiT::FLAG *Bhe* BepD fusion protein	This study

Supplementary Table S3: List of oligonucleotide primers used in this study

Primer	Sequence	Purpose
prKF001	GAGCCGGGATCCTTTTTTCGCTGTGTGAGC	*ΔbepA*_US_fw_BamHI
prKF002	TTTTGGCATTGTTACCTCC	*ΔbepA*_US_rv
prKF003	TTATAAGGAGGTAACAATGCCAAAATAATAAAGTAAA AATTTGCAGGATATTCTTTC	*ΔbepA*_DS_fw
prKF004	GAGCCGTCTAGAATGTAGTTTTATTGCCAGGC	*ΔbepA*_DS_rv_XbaI
prKF005	TATGACAATTCGCAAACCC	sequencing *ΔbepA*_fw
prKF006	TTTATATCCACCAGAACCGG	sequencing *ΔbepA*_rv
prKF007	ATTGGTATAAAAATAAGCGCC	sequencing *ΔbepA*_intern
prKF008	GAGCCGGGATCCAACCTGAGAGAAACACTGATCC	*ΔbepD*_US_fw_BamHI
prKF009	CTTTTTCATGTATGTTTCCTTTC	*ΔbepD*_US_rv
prKF010	TTGAAAGGAAACATACATGAAAAAGGCGATGTAAATA TACATAAACTGTTATC	*ΔbepD*_DS_fw
prKF011	GAGCCGTCTAGATTCGTCTTTACAGCCTTGG	*ΔbepD*_DS_rv_XbaI
prKF012	ATCTGTTTGAGGATAGCACCC	sequencing *ΔbepD*_fw
prKF013	TTTTTTCAGCTTCTTTGCG	sequencing *ΔbepD*_rv
prKF014	TTATTGTATTGCTTTGTGCC	sequencing *ΔbepD*_intern
prKF015	GAGCCGGGATCCATCCTTAATGCTCTTTTATCAATCC	*ΔbepC-I*_US_fw_BamHI
prKF016	CTCTAACATAGGATATCTCCTTAGAGAATAG	*ΔbepC-I*_US_rv
prKF017	AAGGAGATATCCTATGTTAGAGTGTCTATAAATTTCAA TTTTTCAGCC	*ΔbepC-I*_DS_fw
prKF018	AAGAAAGATTTAAGCCGATATGC	*ΔbepC-I*_DS_rv_XbaI
prKF019	ATGCAATGATTACAGCTGACG	sequencing *ΔbepC-I*_fw
prKF020	AATACCTCCCGTGATGGC	sequencing *ΔbepC-I*_rv
prKF021	AAAAATACGGCTCATCAAGG	sequencing *ΔbepC-I*_intern
prKF026	GAGCCGGGATCCAATCACTTTGGAGAAGCG	*ΔbepC*_US_fw_BamHI
prKF027	CTCTAACATAGGATATCTCC	*ΔbepC*_US_rv
prKF028	TCTAAGGAGATATCCTATGTTAGAGACCGGCTAAAAAC TGATATAATTT	*ΔbepC*_DS_fw
prKF029	GAGCCGTCTAGAAAGACGTTCTCTCCTTCTCG	*ΔbepC*_DS_rv_XbaI
prKF030	AAAAAGCGTGTTTTGTTCG	sequencing *ΔbepC*_fw
prKF031	AAGAGCAGCACAAAGAGGG	sequencing *ΔbepC*_rv
prKF032	ATAGTTTCTTCTGATTGTGGGG	sequencing *ΔbepC*_intern
prKF033	GAGCCGGGATCCTTTGGTGAAAATGCTGGG	*ΔbepF*_US_fw_BamHI
prKF034	TTTTTTCATGCCTGTTTCC	*ΔbepF*_US_rv

prKF035	TTGAAAGGAAACAGGCATGAAAAAAACCAGCTAAACT TCATAACCTATTG	*ΔbepF*_DS_fw
prKF036	GAGCCGTCTAGAAAAATTCTAGTTCGTGACCTGC	*ΔbepF*_DS_rv_XbaI
prKF037	TTACTACAGCACCGTTGGC	sequencing *ΔbepF*_fw
prKF038	AGCGTTTTTTCTGGATTGG	sequencing *ΔbepF*_rv
prKF039	TTTCTGAGGAGGTAAGGTGC	sequencing *ΔbepF*_intern
prKF040	GAGCCGGGATCCTACAACACAAACAAGAAAGCG	*ΔbepI*_US_fw_BamHI
prKF041	GTCTCTCATAGATGTTTCCTTTCAC	*ΔbepI*_US_rv
prKF042	GTGAAAGGAAACATCTATGAGAGACTGTCTATAAATTT CAATTTTTCAGC	*ΔbepI*_DS_fw
prKF043	GAGCCGTCTAGAAAGAAAGATTTAAGCCGATATGC	*ΔbepI*_DS_rv_XbaI
prKF044	TTATCAAAACCTCCTAAACAACC	sequencing *ΔbepI*_fw
prKF105	GAGCCGTCTAGATCACTCTGTTTCTCGTCTTGC	*ΔvirD4*_US_fw_BamHI
prKF095	GTATTTCATTGTCTCTTACTTTCG	*ΔvirD4*_US_rv
prKF096	GAGACAATGAAATACAAAAAGTGAAAAATATTC	*ΔvirD4*_DS_fw
prKF106	GAGCCGGGATCCTTGTGTGGGTTTTTGATGC	*ΔvirD4*_DS_rv_XbaI
prKF098	AACAAATCCAGAAATGCG	sequencing *ΔvirD4*_fw
prKF099	TAAGCAGCATCAAATTTTCG	sequencing *ΔvirD4*_rv
prKF100	TGTGAAAATCGTGGTTATGG	sequencing *ΔvirD4*_intern
prKF164	GAGCCGGGATCCAAGAAGGAGATATACAAATGGTGAG	expression *HiBiT-FLAG*_fw_BamHI for pKF027
prKF165	ATTCTTTTTTTTGTCATCGTCATCCTTG	expression *HiBiT-FLAG*_rv for pKF027
prKF166	ATGACAAAAAAAAGAATCATCCATCCCC	expression *bepD*$_{Bta}$_fw for pKF027
prKF167	GAGCCGGTCGACTTACATCGCAAAAGCCATTC	expression *bepD*$_{Bta}$_rv_SalI for pKF027
prMO001	GCGGGATCCAAGAAGGAGATATACAAATGGTGAGC	expression *HiBiT-FLAG*_fw_BamHI for pMO006
prMO010	GATTTTTTTTTTTGTCATCGTCATCCTTGTAATC	expression *HiBiT-FLAG*_rv for pMO006
prMO011	GACGATGACAAAAAAAAAAATCGACCATCCCCTC	expression *bepD*$_{Bhe}$_fw for pMO006
prMO012	GCGGGTACCTTACATACCAAAGGCCATTC	expression *bepD*$_{Bhe}$_rv_KpnI for pMO006

1.3 References supplementary materials

Dehio, M., Knorre, A., Lanz, C., and Dehio, C. (1998). Construction of versatile high-level expression vectors for Bartonella henselae and the use of green fluorescent protein as a new expression marker. *Gene* 215(2)**,** 223-229. doi: 10.1016/s0378-1119(98)00319-9.

Harms, A., Liesch, M., Korner, J., Quebatte, M., Engel, P., and Dehio, C. (2017a). A bacterial toxin-antitoxin module is the origin of inter-bacterial and inter-kingdom effectors of Bartonella. *PLoS Genet* 13(10)**,** e1007077. doi: 10.1371/journal.pgen.1007077.

Harms, A., Segers, F.H., Quebatte, M., Mistl, C., Manfredi, P., Korner, J., et al. (2017b). Evolutionary Dynamics of Pathoadaptation Revealed by Three Independent Acquisitions of the VirB/D4 Type IV Secretion System in Bartonella. *Genome Biol Evol* 9(3)**,** 761-776. doi: 10.1093/gbe/evx042.

Quebatte, M., Dehio, M., Tropel, D., Basler, A., Toller, I., Raddatz, G., et al. (2010). The BatR/BatS two-component regulatory system controls the adaptive response of Bartonella henselae during human endothelial cell infection. *J Bacteriol* 192(13)**,** 3352-3367. doi: 10.1128/JB.01676-09.

Schmid, M.C., Schulein, R., Dehio, M., Denecker, G., Carena, I., and Dehio, C. (2004). The VirB type IV secretion system of Bartonella henselae mediates invasion, proinflammatory activation and antiapoptotic protection of endothelial cells. *Mol Microbiol* 52(1)**,** 81-92. doi: 10.1111/j.1365-2958.2003.03964.x.

Schulein, R., and Dehio, C. (2002). The VirB/VirD4 type IV secretion system of Bartonella is essential for establishing intraerythrocytic infection. *Mol Microbiol* 46(4)**,** 1053-1067.

Schulein, R., Guye, P., Rhomberg, T.A., Schmid, M.C., Schroder, G., Vergunst, A.C., et al. (2005). A bipartite signal mediates the transfer of type IV secretion substrates of Bartonella henselae into human cells. *Proc Natl Acad Sci U S A* 102(3)**,** 856-861. doi: 10.1073/pnas.0406796102.

Sorg, I., Schmutz, C., Lu, Y.Y., Fromm, K., Siewert, L.K., Bogli, A., et al. (2020). A Bartonella Effector Acts as Signaling Hub for Intrinsic STAT3 Activation to Trigger Anti-inflammatory Responses. *Cell Host Microbe* 27(3)**,** 476-485 e477. doi: 10.1016/j.chom.2020.01.015.
